# State and Inter-State Medicine

**Published:** 1883-05

**Authors:** 


### Editorial.

### STATE AND INTER-STATE MEDICINE.

IN our issue of last month, we published the interesting address of Prof. Moses Gunn, of Rush Medical College, delivered at the late Commencement exercises of that institution. Our readers have already enjoyed the opportunity of perusing its pages, which set forth in the clearest and most comprehensive manner the ethical questions which have lately been engaging the attention of medical men. It is unnecessary to go over again the ground there transversed. This exposition of the views of a veteran instructor and practitioner, holding, on the one hand, the conservative opinions on the subject of the code entertained for a generation by the best representatives of regular medicine in the country, and, on the other, acknowledging that respect for and deference to the existing laws of the State which are the requisites of all good citizenship, has already attracted the attention it merits.

The prominence, however, thus given to the *status* and functions of the Illinois State Board of Health, has suggested a view of the results of its operations which is not without further interest. We remark, in passing, that we entertain fort he gentlemen composing this honorable body, no less respect than that distinctly expressed by the distinguished author of the address to which we have referred.

It is claimed by them, and we doubt not justly, that in consequence of their organization and operations, a large number of irregular practitioners have been driven beyond the borders of this State. We do not understand that it is claimed that all such have been thus removed, for even a superficial examination of the daily papers of this city discloses the advertisements of a number of such irregular practitioners, some of whom, we understand, have begun their profitable business in this line, since the passage of the law which, by its provisions, released from its penalties all those who by ten years of irregular practice had secured exemption.

Granting, however, that the operation of the law and the activities of the Board have driven beyond the borders of this State a horde of irregular practitioners, the questions naturally arise, where are they now and what are they doing? If all of the several States of the Union had similar Health Boards with powers equal to those conferred on the Illinois body, the grand result might be that of the equal operation of a national law, affecting all the States of the Union to the same extent. As the matter stands at present, it is well known that there are many States of the Union not provided with Boards of Health. If there are those who wish to know whether quackery and charlatanism are prevalent in such political divisions of the country, their desire may be readily satisfied. We refer any enquirers on this point to the late address of the President of the Missouri State Medical Society, whose revelations were made the theme of some very pertinent editorial comments in a recent number of the *N. Y. Medical Record.* A reference to the original pages of this address, however, will demonstrate clearly that even extravagant language would fail to properly characterize the abject ignorance, the depravity and even the villainy of the impostors who have settled upon the unfortunate people of the State of Missouri in a swarm worse than that of the traditional Egyptian locusts. This may prove a startling and fearful exception to the rule, but it certainly suggests to the liberal-minded to ask to what extent the unequal operation of law is working a serious injury to some parties of this country. However much the present tariff may be claimed as a strictly American system of protection, it is firmly established that free-trade is to be the rule forever in at least all commerce between the States. The necessity for the equal operation of law upon all citizens in all districts is nowhere better recognized than in Illinois, which, by statute, has prohibited special legislation for its own citizens. That which is good and useful for one part of the country should not be less desirable for all others.

If the operation of State laws of the kind to which we refer is really working an injury to our sister States, one naturally inquires, what is the remedy? If it be responded, the several States should protect themselves by organizing their own Board of Health, the improbability, not to say impossibility, of the passage of laws with exactly similar provisions for regulating practice in so many differently organized political divisions of the country, becomes at once apparent. Shall we, in despair of such an issue, proceed at once to destroy the effectiveness of our own law, with a magnanimity and unselfishness unequaled save in the pages of the Light of Asia? Surely there is a broader view to be taken of the whole matter.

What has been actually attempted and accomplished is admittedly imperfect in result, limited as to scope, unequal in action and open in many respects to adverse criticism. With such limited powers as it possessed, we are willing to admit that the Illinois Board entered upon its task with enthusiasm and devoted itself to its work with energy. But the need for something better and broader is apparent. Either the national congress must pass such general laws for the regulation of the practice of medicine in the United States as shall operate equally in all parts of the country, or its regularly educated physicians must take steps to protect themselves. As a rule they have fared badly when they have asked protection of politicians, law-makers and legislators. Their best and broadest results have been accomplished either in the absence of all laws specially affecting them or in face of the worst. Their best representatives have constituted a body of men the peers of the profession in any country of the world where the most stringent regulations limit admission to their ranks and govern all who are admitted. Their worst representatives are not the fruit but the by-products of their best efforts.

After all, the system is part and parcel of one huge crudity of magnificent resources and inferior methods. This is a country whose laws are so defective that it is actually possible for a contingency to arise in which there would be a doubt as to who is the chief magistrate of the nation. Can any wonder, then, that there are gross irregularities in all legislation relating to the practice of medicine?

